# A patient allergic to multiple chemically unrelated dyes

**DOI:** 10.1111/cod.13705

**Published:** 2020-10-07

**Authors:** Tirza Blom, Edith M. de Boer, Thomas Rustemeyer

**Affiliations:** ^1^ Department of Dermatology Amsterdam University Medical Center Amsterdam The Netherlands

**Keywords:** allergic contact dermatitis, case report, textile dye dermatitis

## CASE REPORT

A 63‐year‐old woman visited the allergology department with dermatitis of the face and forearms, occurring after wearing a new unwashed blue synthetic jacket (90% polyamide and 10% down) in the rain. In the past she experienced dermatitis on the breasts after wearing an unwashed new black bra. She had no atopic personal or family history. Patch testing, performed with the European baseline, local supplementary, and perfume series according to the ESCD guideline,^1^ showed positive results for Disperse Blue 106, textile dye mix, fragrances, and preservatives. Additional testing with textile dyes from Chemotechnique Diagnostics (Vellinge, Sweden) (1% pet.) showed positive patch test reactions to acid, basic, disperse, and reactive dyes, namely, A*cid* Red 118, *Basic* Red 46, *Disperse* Blue 35, 106, 124, and 153, as well as Disperse Brown 1, Orange 3, Red 17, and Yellow 3. Moreover, *Reactive* Black 5, Blue 238, and Red 123, 228, and 238 elicited positive patch test reactions. These were not considered to represent an angry back syndrome. These positive results were considered clinically relevant because of a clear correlation between wearing some unwashed garments and a flare‐up of dermatitis. Wearing white or light‐colored clothes and washing new clothes before wearing was recommended. After pimecrolimus cream 10 mg/g treatment and eliminating all allergens, the skin lesions disappeared. During the next 6 months short‐lasting eczema occurred after shopping for new clothes.

## DISCUSSION

An Italian multicenter study observed concomitant reactions among textile dyes and/or finishing resins in 50.0% of 277 patients.^2^ The multiple sensitizations were explained by cross‐sensitization between similar chemical dye structures. In case of sensitization to more than one reactive dye, all compounds shared the same reactive group.^3^ In our case, Reactive Blue 238 and Reactive Black 5 share a vinyl sulphonyl moiety. The reactive group of other reactive dyes tested positive in this case are unknown. Disperse Blue 106 and 124 have similar chemical structures. Cross‐sensitization between Disperse Blue 106 and 124 occurs most often.^2^

Simultaneous positive reactions to different chemical dye groups resulting in co‐sensitization can be attributed to the mixture of dyeing agents used to dye a fiber blend in textiles.^4^ For example, Red and Yellow dyes are used to achieve different shades of blue. Another explanation could be that the purity of the dyes used for patch testing was insufficient, resulting in extensive allergic reactions. Dyes are not routinely tested in most countries, and extensive testing is exceptional; therefore co‐sensitization and cross‐sensitization is researched only in small samples.^5^ Few cases are described with sensitization to chemical unrelated dyes.^3^, ^6^ Positive reactions to all four different chemical dye types (our case) have not been described before.

Health care professionals should be aware that multiple allergies to various types of dyes are possible. In patients with multiple positive reactions to chemically (un)related dyes, angry back syndrome, cross‐sensitization, and co‐sensitizations should be considered. Curiosity and routinely additional testing in patients reacting to a dye(mixture) may add insights into the threats of a colorful life. Collaboration between doctors and dyeing (textile) industries is required.

## CONFLICT OF INTEREST

The authors declare no potential conflict of interest.

## AUTHOR CONTRIBUTIONS

**Tirza Blom:** Conceptualization; investigation; project administration; resources; writing‐original draft; writing‐review and editing. **Edith de Boer:** Conceptualization; investigation; resources; supervision; writing‐review and editing. **Thomas Rustemeyer:** Conceptualization; investigation; resources; supervision; writing‐review and editing.

## References

[1] Bruze M , Conde‐Salazar L , Goossens A , Kanerva L , White IR . Thoughts on sensitizers in a standard patch test series. The European Society of Contact Dermatitis. Contact Dermatitis. 1999;41(5):241‐250. 10554056 10.1111/j.1600-0536.1999.tb06154.x

[2] Lisi P , Stingeni L , Cristaudo A , et al. Clinical and epidemiological features of textile contact dermatitis: an Italian multicentre study. Contact Dermatitis. 2014;70(6):344‐350. 24392992 10.1111/cod.12179

[3] Moreau L , Goossens A . Allergic contact dermatitis associated with reactive dyes in a dark garment: a case report. Contact Dermatitis. 2005;53(3):150‐154. 16128754 10.1111/j.0105-1873.2005.00663.x

[4] Hatch KL , Maibach HI . Textile fiber dermatitis. Contact Dermatitis. 1985;12(1):1‐11. 3884253 10.1111/j.1600-0536.1985.tb01030.x

[5] Seidenari S , Mantovani L , Manzini BM , Pignatti M . Cross‐sensitizations between azo dyes and para‐amino compound. A study of 236 azo‐dye‐sensitive subjects. Contact Dermatitis. 1997;36(2):91‐96. 9062744 10.1111/j.1600-0536.1997.tb00420.x

[6] Perez‐Crespo M , Silvestre JF , Lucas A , Ballester I . Co‐sensitivity to disperse and reactive dyes. Contact Dermatitis. 2009;60(4):223‐225. 19338592 10.1111/j.1600-0536.2008.01409.x

